# Effectiveness of ertapenem for treatment of infections in children: An evidence mapping and meta-analysis

**DOI:** 10.3389/fped.2022.982179

**Published:** 2022-10-12

**Authors:** Ruiqiu Zhao, Xiaoru Long, Jiangxia Wang, Jing Zhu, Cong Liu, Tingting Shang, Zhenzhen Zhang, Engels Obi, Lynda Osadebe, Yue Kang, Jie Liu, Xiaodi Chen, Hongmei Xu

**Affiliations:** ^1^Chongqing Key Laboratory of Child Infection and Immunity, Chongqing Key Laboratory of Pediatrics, Ministry of Education Key Laboratory of Child Development and Disorders, Department of Infectious Diseases of Children’s Hospital of Chongqing Medical University, National Clinical Research Center for Child Health and Disorders, China International Science and Technology Cooperation Base of Child Development and Critical Disorders, Chongqing, China; ^2^Merck & Co., Inc., Rahway, NJ, United States; ^3^MRL Global Medical Affairs, MSD China, Shanghai, China

**Keywords:** children, infection, ertapenem, efficacy, safety

## Abstract

**Objectives:**

To assess and summarize current evidence on the effectiveness and safety of ertapenem for treatment of childhood infections, in consideration of high infection prevalence in children and wide use of ertapenem.

**Methods:**

The following 8 databases were searched on 13th May 2021: Web of Science, Embase via Ovid SP, PubMed, The Cochrane Library (CENTRAL), Chinese BioMedical Literature Database (CBM), China National Knowledge Infrastructure (CNKI), VIP and Wanfang. The primary outcome was treatment success rate. Risk ratios (RRs) and 95% confidence interval (CI) were estimated using random-effect models. Subgroup analysis was conducted where heterogeneity was found.

**Results:**

Fifteen studies (8 randomized controlled trials, 1 observational comparative study, and 6 before and after studies) involving 2,528 patients were included in the final review. Ertapenem had similar treatment success rates with β-lactam antibiotics [relative risk (RR) = 1.08, 95% CI: 0.99–1.19]. In a subgroup analysis, similar efficacy (RR = 1.08, 95% CI: 0.97–1.20) between ertapenem and other carbapenems. Compared with β-lactam antibiotics, ertapenem did not increase the risk of any adverse events (RR = 1.02, 95%CI: 0.71–1.48), drug-related diarrhea (all non-Asian children, RR = 0.62, 95%CI: 0.31–1.25), or injection site pain (all non-Asian children, RR = 1.66, 95%CI: 0.59–4.68). Subgroup analysis showed no obvious difference between ertapenem group and carbapenems or non-carbapenems group on risk of adverse events.

**Conclusion:**

Our findings suggest that ertapenem is effective and safe in treatment for children with infection. Further comparative real-world data is needed to supplement clinical evidence on the overall benefits of ertapenem in this population.

## Introduction

Bacterial infections pose a major threat to children’s health with numerous children dying from bacterial infections every year (1–3). Children with infectious diseases are commonly encountered in primary care settings (4). Perforated appendicitis (5–7), severe community-acquired pneumonia (CAP) (8), complicated intra-abdominal infections (cIAI), acute leukemia secondary to infections (9, 10) and complicated urinary tract infections (cUTI) (11, 12) are considered truly life-threatening for children. In addition to harms directly resulted from infection itself, infection can also lead to serious consequences such as leukemia and secondary renal function decline (13–15). Therefore, effective anti-infective therapy in children is of great significance in clinical practice.

Previous evidence has shown that the etiology and pathophysiology of bacterial infections in children, as well as the metabolism and tolerance of drugs, are different from that in adults (16, 17). The main treatment methods of childhood infections include surgery, antibiotics including beta-lactam, carbapenem, macrolide and other drugs, and supportive treatments. However, the routine use of broad-spectrum antibiotics, especially carbapenem, for childhood infections is not recommended (16) under many circumstances. Ertapenem, a 1β-methyl carbapenem antibiotic that has good antibacterial activity and pharmacokinetic properties and can be administered once a day, is mainly metabolized in the kidneys but rarely in the liver (18). Therefore, it can be used safely in adult patients with moderate to severe renal function decline receiving hemodialysis. Ertapenem achieves its bactericidal effect by combining with penicillin binding proteins to inhibit cell wall synthesis.

Ertapenem was first approved in the United States in 2001 for the treatment of CAP and cIAI (19) in particular. It was then approved in 2005 for the treatment of childhood infections. After nearly two decades of clinical application, experience in the use of ertapenem has piled up, especially in the treatment of childhood infections. However, studies assessing the effectiveness and safety of ertapenem have focused primarily on the adult population (20) and no systematic review in children is available. Taking the high infection prevalence in children (5, 21, 22) worldwide into consideration, there is a need for more recent evidence evaluating outcomes associated with use of ertapenem for treatment of infections in children. In this study, we conducted an evidence mapping and meta-analysis of studies assessing effectiveness and safety of ertapenem for treatment of infections in children. Study findings should provide important data to help inform infectious disease management and appropriate use of antibiotics in this vulnerable population.

## Materials and methods

This study applied a methodology combining both evidence mapping and meta-analysis, based on recommendations from the Cochrane handbook and PRISMA statement (23).

### Search strategy

A full review of literature was undertaken to identify all relevant studies investigating the effectiveness of ertapenem or ertapenem combinations on pediatric patients with bacterial infectious diseases. To ensure that all relevant studies were identified, a comprehensive search of online biomedical databases was conducted in May 2021 including Web of Science, Embase, PubMed, The Cochrane Library (CENTRAL), Chinese BioMedical Literature Database (CBM), China National Knowledge Infrastructure (CNKI), VIP and Wanfang (Supplementary Appendix Search Strategy).

### Criteria for study inclusion/exclusion

We included peer-reviewed studies of pediatric patients aged between 3 months and 18 years with bacterial infectious diseases and receiving ertapenem or ertapenem combinations without limitations on dosage or frequency and treatment duration. Types of study designs included randomized controlled trials (RCTs), non-randomized controlled trials (non-RCTs), and observational studies (cohort studies, case-control studies, cross-sectional studies, before and after studies). Both English and Chinese language publications were eligible for inclusion.

### Study selection

Two independent reviewers screened search results. All potentially relevant citations were requested and inspected in detail via the full text paper. Disagreements were resolved by discussion, with the assistance from a third party if necessary. A PRISMA flow diagram was constructed to show the full study-selection process (23).

### Outcomes

The primary outcome was treatment success rate, defined as the proportion of patients who completed the treatment with evidence of success (cured) and based on the results yielded from original studies. Secondary outcomes included length of stay (defined as the number of days of hospitalization from receiving the intervention to being discharged from the hospital), mortality rate (defined as the number of all-cause deaths reported for the cohorts of interest during treatment or follow-up, as reported by the source studies), incidence of serious drug-related clinical and/or laboratory adverse events (as defined in original studies), and study withdrawals due to adverse events.

### Data extraction

After screening and determining eligibility, data from each study were extracted independently by two reviewers using a standardized data abstraction form. Study elements abstracted included first author of study, methods (location setting, study design, inclusion and exclusion criteria, length of follow-up, blinded or not), participants (diagnosis, age, sex, sample size), interventions (number of study arms, description, frequency, dosage, duration), outcomes (pre-specified primary and secondary outcomes, other outcomes that are defined as reported by original studies and not listed among pre-specified outcomes) and results (dichotomous result, continuous result).

### Data synthesis

For evidence mapping, we grouped and summarized studies by publication year, publication country, comparators based on antibiotic class as applied in prior literature (24), and study design categorized as RCT, non-RCT, observational comparative study, before and after study.

### Risk of bias assessment

For RCTs included in the evidence synthesis, the validity of individual trials was assessed using the Risk of Bias instrument (25), endorsed by the Cochrane Collaboration. For the non-RCTs and observational studies included, we evaluated the quality of studies using the Newcastle-Ottawa Quality Assessment Scale (NOS) (26). When ≥ 10 studies were included to investigate a particular outcome, funnel plots were used to assess small study effects (27).

### Quality of evidence

Based on the Grading of Recommendations Assessment, Development and Evaluation (GRADE) approach (28), the quality of evidence was graded as being of high certainty, moderate certainty, low certainty, or very low certainty.

### Statistical analyses

Dichotomous outcome data were summarized using risk ratios (RRs) and continuous outcome data summarized using mean differences (MDs), both with 95% confidence intervals (CIs). Skewed data were identified and diagnosed from means and standard deviations of pre-determined outcomes. We assessed, with positive measurements, that where “the mean is smaller than twice the standard deviation the data is likely to be skewed” (29, 30). Skewed data was narratively reported.

In the current meta-analysis, we did not reproduce data if more than 50% was unaccounted for. For binary data where loss ranged between 0 and 50%, we presented such data on a “once-randomized-always-analyze” basis (an intention-to-treat analysis) assuming that all missing data from intervention or control group experienced the events. For continuous data, we only reproduced data where between 0 and 50% of patients completed the study up to that point. Where standard deviations were not reported, we attempted to obtain them from authors and if unsuccessful, applied methods for imputation of standard deviations outlined in the Cochrane Handbook (30). Studies having data with attrition rates greater than 20% were marked as having a high risk of bias, and sensitivity analyses conducted rather than excluding such studies. Potential reasons for heterogeneity, if identified, were explored. Data was synthesized (RRs and MDs) using a fixed-effect method for all analyses. Where heterogeneity was found, data was pooled using random-effect models and subgroup analysis conducted accordingly. When the source inducing heterogeneity could not be figured out, the data was synthesized in a narrative fashion.

Given potential heterogeneity that may arise in patient populations on intervention drugs, we conducted subgroup comparisons including ertapenem vs. beta-lactam antibiotics (carbapenems), ertapenem vs. beta-lactam antibiotics (non-carbapenems), Asian vs. non-Asian patient population, different bacterial infection sites (cIAI, cUTI), post-infection cough and CAP. Furthermore, we also undertook a sensitivity analysis for studies rated as having high risk of bias.

## Results

### Study selection

**FIGURE 1:**
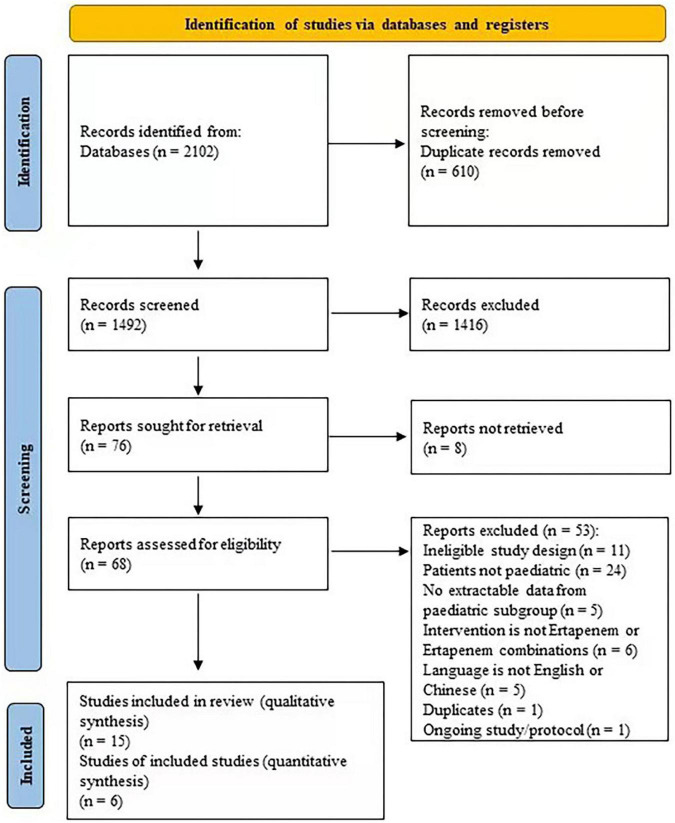
Study flow diagram.

We identified 2,102 articles in the databases searched. After removing duplications and non-eligible articles, 15 articles were included in the present study (Figure 1 and Table 1) (6, 31–44).

**TABLE 1:** Characteristics of included studies.

References	Study design	Sample size	Male/ Female	Mean age	Age (Min-Max)	Diagnosis	Intervention/ Comparator(s)	Outcome reported	Key findings reported in paper
Arguedas et al. (31)	RCT	404	162/241	NR	3 months-17 years	Complicated urinary tract infection (cUTI) Community-acquired pneumonia (CAP) Skin and soft-tissue infection (SSTI)	Ertapenem/ ceftriaxone	Length of stay/clinical AE/laboratory AE	No significant difference on reported outcomes
Arnold et al. (32)	RCT	82	41/41	Ertapenem: 12.3 ± 3.6 years ertapenem + amoxicillin-clavulanate: 10.1 ± 3.6 years	4–17 years	Perforated appendicitis	Ertapenem/ Ertapenem + amoxicillin-clavulanate	Length of stay/clinical AE	No significant difference on reported outcomes
Dalgic et al. (33)	RCT	107	74/33	Ertapenem: 118.62 ± 36.01 months; ampicillin + gentamicin + metronidazole: 118.62 ± 36.01 months	3 months-17 years	Perforated appendicitis	Ertapenem/ Standard triple (ampicillin + gentamicin + metronidazole)	Length of stay/clinical AE	No significant difference on reported outcomes
Jin (34)	RCT	76	44/32	NR	2–11 years	Acute leukemia with bacterial infection	Ertapenem/ Imipenem + statin	Treatment success rate/ clinical adverse events	No significant difference on reported outcomes
Pogoreliæ et al. (35)	RCT	80	62/18	NR	5–17 years	Perforated Appendicitis with diffuse peritonitis	Ertapenem/ Gentamicin + metronidazole	Treatment success rate/ length of stay/clinical AE	Significant, favor Ertapenem on length of stay; No significant difference on other reported outcomes
Yi et al. (36)	RCT	100	54/46	Ertapenem: 6.09 ± 1.49 years	NR	severe Community-acquired pneumonia (CAP)	Ertapenem/ Meropenem	Treatment success rate/length of stay/clinical AE	No significant difference on reported outcomes
Wirth et al. (37)	RCT	451	277/174	12.04 years	3 months-17 years	Complicated Intra-abdominal Infections (cIAI)	Ertapenem + amoxicillin + clavulanate/ moxifloxacin	Treatment success rate/ mortality rate/clinical AE	Significant, favor Ertapenem combination on treatment success rate and clinical AE
Yellin et al. (6)	RCT	112	40/65	NR	2–17 years	Complicated intra-abdominal infections (cIAI) or acute pelvic infections (API)	Ertapenem/ ticarcillin + clavulanate	Treatment success rate/ mortality rate/clinical AE/ laboratory AE	No statistical significance was reported. But a conclusion of generally safe and efficacious on Ertapenem compared to control group
Abdel-Rahman et al. (38)	Before and after study	84	42/42	<2 years (*n* = 41): 1.0 ± 0.6 years 2–12 years (*n* = 28): 6.7 ± 3.4 years >12 years (*n* = 11): 14.3 ± 0.9 years	3 months-16 years	Lower respiratory tract infection (*n* = 23), skin/skin structure infection (*n* = 15), upper respiratory tract infection (*n* = 14), pyelonephritis (*n* = 8), appendicitis (*n* = 4), central line infection (*n* = 3), postoperative fever (*n* = 3), septic arthritis (*n* = 2), fever with neutropenia (*n* = 2), urinary tract infection (*n* = 2), osteomyelitis (*n* = 1), sepsis (*n* = 1), gastrointestinal infection (*n* = 1), endocarditis (*n* = 1), unclear reason (*n* = 4)	Ertapenem/NA	Clinical AE	Abdel-Rahman 2010 is a pK study of ertapenem. Three children had adverse events including 2 cases had nausea, and 1 case had injection site reaction.
Blanco et al. (39)	Before and after study	544	NR	NR	NR	Perforated appendicitis	Ertapenem/NA	Length of stay	The average length of stay after ertapenem treatment, decreased from 6.4 ± 5.3 days to 4.5 ± 2.9 days (*p* < 0.0001).
Dalgic et al. (40)	Before and after study	50	20/30	38.6 ± 36.9 months	6 months-13 years	Complicated urinary tract infection (cUTI) caused by ESBL-producing microorganisms (including: no urinary abnormalities, neurogenic bladder, obstructive uropathy, bladder exstrophy, traumatic urethral disruption)	Ertapenem/NA	Treatment success rate/length of stay/symptom remission time/clinical AE	Clinical cure and bacteriological eradication in the urine were achieved in all patients. The average length of hospital stay was 10.28 ± 3.98 days (range 7–21 days). Urine culture was negative 3.3 ± 0.7 days after starting ertapenem treatment. None of the patients had any clinical or laboratory adverse events nor a persistent infection. Secondary infections were diagnosed in 4 patients, including 2 fungal infections.
Xinshun and Rong (41)	Before and after study	121	70/51	3.7 ± 0.8 years	3 months-14 years	Pneumonia, bronchitis, urinary tract infections, Sepsis, appendicitis and appendicitis abscess, abdominal infection, osteomyelitis, soft tissue infections, bacterial infection of underlying disease	Ertapenem/NA	Treatment success rate/clinical AE	The clinical cure rate is 92.6% (112/121), and 8 out of 121 patients occurred with diarrhea.
Karaaslan et al. (42)	Before and after study	77	16/61	76.6 ± 52 months	3 months-17 years	Complicated urinary tract infections caused by ESBL-producing bacteria	Ertapenem/NA	Treatment success rate/length of hospital stay/clinical AE/laboratory AE	The culture of all patients resulted to be negative. There was no serious drug-related clinical or laboratory adverse effect occurred. Two drug-related AEs were observed, with one patient having a mildly elevated level of alanine aminotransferase and another patient developing a short-term maculopapular rash.
Rutkoski and Gaines(43)	Before and after study	144	79/65	9 years	2–18 years	Perforated appendicitis had appendectomy	Ertapenem/NA	clinical AE	This is an abstract, the study reported that infectious complications included intra-abdominal abscess in 35 (24%) and wound infection in 5 (3.5%).
Filip et al. (44)	Observational comparative study	96	56/40	Ertapenem: 10 years and 8 months ceftriaxone + gentamycine: 9 years and 3 months	NR	Acute perforated appendicitis and subsequent peritonitis (APAP)	Ertapenem/ ceftriaxone + gentamycin	Length of stay	No significant difference on reported outcomes

### Risk of bias assessment

Two out of 8 RCTs, were rated as having a high risk of bias due to non-blinding of participants and personnel, and potential conflict of interest, such as commercial funding (31, 33). The other 6 studies were rated as having moderate risk of bias due to moderate risk of non-blinding outcome assessment, high attrition rate or selective reporting (6, 32, 34–37). One observational comparative study was rated as seven stars by NOS scale, which means a high quality. The retrospective study design and insufficient length of follow-up compromised the overall quality of this study (Supplementary Appendix Table 1).

### Study characteristics

**FIGURE 2:**
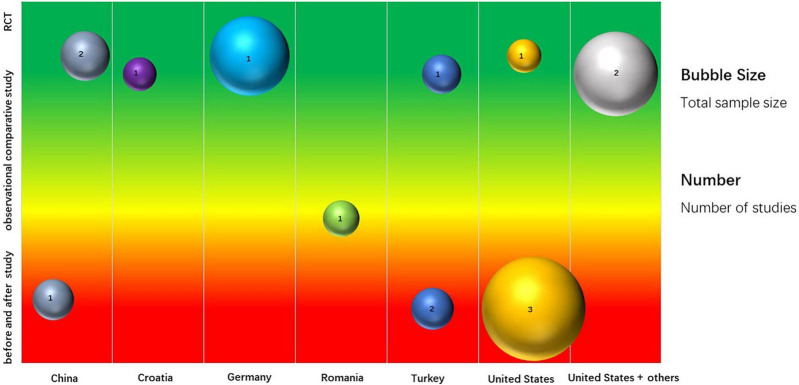
Mapping of included studies.

A total of 15 studies were included based on inclusion/exclusion criteria (Table 1). These included 8 RCTs comparing single or combined ertapenem therapy with other single antibiotics (such as ceftriaxone, meropenem, and moxifloxacin) or with combined therapies (such as ticarcillin + clavulanate, gentamicin + metronidazole) (6, 31–37), 1 observational comparative study (ertapenem vs. ceftriaxone + gentamycin) (44), and 6 before and after studies (38–43). These studies included 2,528 patients from 9 countries (Figure 2 and Supplementary Appendix Table 2). Four studies were carried out in the United States (854 patients), 3 each in China (297 patients), and Turkey (234 patients), 1 each in Croatia (80 patients), Germany (451 patients), Romania (96 patients), and 2 in a multinational collaboration (516 patients total from the United States, Spain, Mexico, and Brazil).

Twelve studies reported children between 3 months to 18 years old (the other 3 studies did not report age). Among these patients, perforated appendicitis (*n* = 1,053), cIAI (*n* = 566), CAP (*n* = 281), and skin and soft-tissue infections (SSTI) (*n* = 140) were predominant (Table 1). In studies comparing outcomes for patients receiving ertapenem, single or combination therapy, vs. other drugs, 8 studies reported treatment success rates (6, 34–37, 40–42), 9 studies reported on length of stay (31–33, 35, 36, 39, 40, 42, 44), and 2 studies reported on mortality rates (6, 37). Twelve studies (6, 31–38, 40–42) reported clinical adverse events, of which 4 studies (6, 31, 34, 36) had data eligible for meta-analysis and 2 studies (6, 31) reported laboratory adverse events. Details of outcome findings of each individual study are listed in Table 1.

### Meta-analysis of ertapenem vs. ß-lactam antibiotics

**FIGURE 3:**
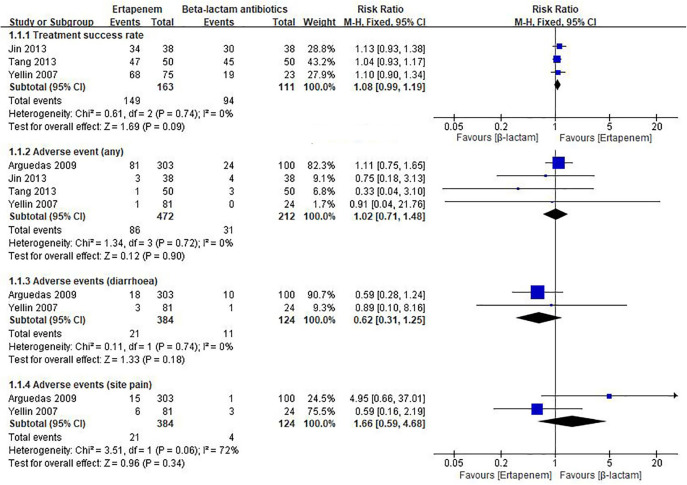
Meta-analysis for ertapenem vs. β-lactam antibiotics in children.

Five studies compared Ertapenem vs. β-lactam antibiotics in children (6, 31, 33, 34, 36). As shown in Figure 3, pooled result indicates compared to β-lactam antibiotics, ertapenem had similar treatment success rate to β-lactam antibiotics (RR = 1.08, 95% CI: 0.99–1.19, moderate quality of evidence, Supplementary Appendix Table 3). In a study involving Asian children, there was no difference in length of hospital stay between the ertapenem group and a standard triple therapy group (MD = 0.44, 95% CI: −0.5 to 1.38, low quality of evidence, Supplementary Appendix Table 3). Meta-analysis of 4 studies showed little to no difference between ertapenem and β-lactam antibiotics groups (see Figure 3) in risk of any adverse events (RR = 1.02, 95% CI: 0.71–1.48, moderate quality of evidence, Supplementary Appendix Table 3). Meta-analysis of 2 studies showed that compared to β-lactam antibiotics, ertapenem had similar injection site pain rate to β-lactam antibiotics (all non-Asian children, RR = 1.66, 95% CI: 0.59–4.68, very low quality of evidence, Supplementary Appendix Table 3), in addition did not increase the risk of drug-related diarrhea (all non-Asian children, RR = 0.62, 95% CI: 0.31–1.25, low quality of evidence, Supplementary Appendix Table 3).

### Subgroup analyses

In a subgroup analysis of 2 studies (*N* = 176, Asian children), there was no difference in the risk of adverse events between ertapenem and other carbapenems (RR = 0.57, 95% CI: 0.17–1.87), and similar efficacy (RR = 1.08, 95% CI: 0.97–1.20). Two studies including 508 children (all non-Asian) also reported no difference in the risk of adverse events between ertapenem and non-carbapenems (RR = 1.11, 95% CI: 0.75–1.64) (Supplementary Appendix Figure 1A).

Whether the patient population comes from Asia or not had no impact on the efficacy and safety of ertapenem vs. B-lactam antibiotics (Supplementary Appendix Figure 1B). Two studies including Asian children (RR = 1.08, 95%CI: 0.97–1.20) and 1 study including non-Asian children (RR = 1.10, 95%CI: 0.90–1.34) showed no difference in treatment success rate (TSR) between ertapenem and β-lactam antibiotics. Also, there was no difference in the risk of adverse events between ertapenem and β-lactam antibiotics, in Asia or in non-Asia (RR = 1.11, 95% CI: 0.75–1.64).

Moreover, the site of bacterial infection did not impact efficacy and safety comparisons between ertapenem and B-lactam antibiotics (Supplementary Appendix Figure 1C). Though each specific result came from single study, when treating CAP, cIAI, acute leukemia with bacterial infection or mixed infection, ertapenem showed similar efficacy and safety compared with β-lactam antibiotics.

## Discussion

In children suffering from bacterial infection, a reasonable choice of antibiotic treatment is critical (45). Given their lower weight, immature liver and renal function and potential drug-related adverse events, the use of antibiotics in children is significantly different from that in adults (46). Antibiotics with a narrow antibacterial spectrum and thus lower likelihood of adverse events, such as ertapenem (47), have been used for treatment of childhood infections, and after nearly two decades of clinical application, experience in the use of ertapenem in children have accumulated. However, studies assessing the effectiveness and safety of ertapenem have focused primarily on the adult population (20) and no systematic review in children is available. Given the high prevalence of bacterial infections in children (5, 21, 22), a review of evidence evaluating outcomes associated with use of ertapenem for treatment of infections in children would help inform clinical practice and disease management.

In the present study, we summarized 15 studies on ertapenem therapy in children with infections. Approximately, 26.7% (4/15) of included studies were conducted in the United States with less than 75% of the overall patient population coming from China, Turkey or other regions of the world. Across these studies, we found no significant difference in treatment success rate, length of hospital stays, and adverse events between ertapenem therapy and other therapies, including meropenem and imipenem. However, the results were imprecise with wide CIs (Figure 3) due to insufficient sample size. Nevertheless, our results showed trends of favorable effect on treatment success rate and lower occurrence of drug-related diarrhea in the ertapenem group although not statistically significant. Subgroup analysis also showed no significant difference between ertapenem and other therapies in treatment success rate, length of stay, and adverse event in children from different regions, or children with different infection sites.

To the best of our knowledge, the present study is the first systematic review and meta-analysis of ertapenem specifically in pediatric patients. In a previous meta-analysis that included pediatric and adult patients, the authors concluded that ertapenem had similar efficacy and safety as ceftriaxone for the treatment of complicated infections, such as CAP, cUTI, and cIAI. The authors opined that ertapenem was an appealing option for the treatment of complicated infections (48). However, there was only one study on pediatric patients included in that meta-analysis and consequently, no subgroup analysis on pediatric patients was conducted (48). Findings from the current study are consistent with the previous meta-analysis. The current study showed that, with regards efficacy, ertapenem was comparable to other commonly used antibiotics in children with cUTI, CAP, skin, and soft-tissue infection, perforated appendicitis, cIAI, or acute pelvic infection.

The results of a study included in the current research showed that for immunosuppressed patients (leukemia), the effectiveness of ertapenem was comparable to other drugs (34). In clinical practice, infection is the main complication secondary to blood cancers in children due to immune suppression from treatment and cancer invasion (49). Besides, most anti-tumor medication treatment and immunosuppressives have significant side-effects on liver, renal and hematopoietic function. Therefore, strong and safe antibiotics are needed for these children. Based on findings in the current study, ertapenem is a reasonable potential choice in children with weak immunity.

In the current study, we found little to no difference between ertapenem and β-lactam antibiotics and other carbapenems in risk of adverse events. Ertapenem is a relatively safe antibiotic which metabolizes mostly in the kidneys and rarely in liver, rendering it safe in patients with liver diseases and effective for patients with urinary tract infection (UTI) (19). This is important to note given UTI is the most common type of bacterial infection among children under 2 years old (11, 50). The guidelines often recommend oral antibiotics for children with UTI (11), while in clinical practice it is difficult to administer oral medication in some children. In such conditions, parenteral ertapenem administration twice a day is a reasonable choice. Due to potential severe toxicity, many anti-bacterials have the poor safety profiles (51, 52). Compared to other carbapenem antibiotics (i.e., imipenem and meropenem), ertapenem has a long half-life and could be administered once a day (18). Since group 1 and 2 carbapenems have the similar efficacy and safety, group 2 carbapenem use, but not ertapenem use, is associated with imipenem-resistant P. aeruginosa (53), prescribing ertapenem is appropriate in the treatment of children with low risk of non-fermenters infections. This is especially for children that do not present with risk factors for Pseudomonas aeruginosa respiratory tract infections given ertapenem has low anti-Pseudomonas aeruginosa activity (54).

Limited results from studies (6, 35) included in the present study showed that ertapenem may help shorten the length of hospital stay, which improves the compliance of treatment, reduces the unnecessary consumption of medical resources and the risk of secondary infection in children. With a benzyl group in its molecular structure, ertapenem has a longer half-life of (4.9 ± 5.7) h and lower clearance rate than imipenem and can be used once a day in adult patients (19). In some studies included in this meta-analysis, ertapenem was also used once a day in some children over the age of 13 years (31, 35, 40). These results support feasibility of a once daily administration of ertapenem, which is convenient for both guardians and children.

As recorded in the present study, evidence supporting the use of ertapenem in children, especially the tailoring of dosage and duration based on the specific disease with which children are diagnosed, remains unclear. Future studies should examine ertapenem’s efficacy and safety for children at different age groups, and compared to antibiotics including piperacillin + clavulanic acid, cefoperazone, sulbactam. Future studies should also examine ertapenem’s effectiveness and safety when used for treatment of bloodstream infection, biliary system infection, infectious endocarditis and gastrointestinal infection, and cost-effectiveness of ertapenem treatment for children with infection compared with other antibiotics.

Our study has some strengths. This study covered common types of infection in children, including respiratory tract infection, UTI, skin and soft-tissue infection, and appendicitis. Therefore, findings regarding ertapenem effectiveness from the current study provide reference of wide range of use for pediatric clinicians. Our study also has certain limitations. First, the number of studies included and their sample sizes are relatively small. As described before, though this study included 15 studies, only 2–4 studies were eligible for meta-analysis of specific outcomes, resulting in evidence with low power. The children included in the present study mainly came from USA, Germany, China, and Turkey, all with less than 1,000 participants, leading to a relatively small sample size for systematic review and meta-analysis. Furthermore, subgroup analysis of each specific outcome only came from a single study which impact generalizability to real-world clinical practice. Second, heterogeneity may exist due to the differences regarding study patients, interventions, and controls. Third, there is potential publication bias as the inclusion of non-RCTs together with RCTs may lower the overall quality of evidence. Specifically, subgroup analysis revealed that evidence of use of ertapenem to treat CAP, cIAI, cUTI, PIC among Asian population was insufficient, and that comparison of ertapenem with aminoglycosides was also insufficient. As for outcome assessment including length of hospital stay, mortality and adverse events, comparison of ertapenem with other drugs or regimens was also insufficient.

In conclusion, ertapenem is a viable antibacterial choice for children with infection. Further RCTs are warranted in the future to provide more valid evidence supporting the use of ertapenem in children.

## Data availability statement

The original contributions presented in this study are included in the article/Supplementary material, further inquiries can be directed to the corresponding author.

## Author contributions

HX, EO, LO, JL, and YK: conception and design. XL, LO, and YK: data curation. XL, JW, and JZ: investigation. EO: formal analysis. HX, JL, and RZ: supervision. EO, JL, and XC: resources management. RZ: manuscript writing. All authors have read and approved the final manuscript.

## Supplementary material

Supplementary Appendix Figure 1 Ertapenem vs. beta-lactam antibiotics (subgroup). **(A)** Ertapenem vs. carbapenems and ertapenem vs. non-carbapenems. **(B)** Asian vs. non-Asian patient population. **(C)** Different bacterial infection sites (cIAI and cUTI), post-infection cough, and CAP.
