# Supplementary material for: Effectiveness of ertapenem for treatment of infections in children: An evidence mapping and meta-analysis
Source: Front Pediatr. 2022 Oct 12;10:982179. doi: 10.3389/fped.2022.982179 (PMC9620802; doi:10.3389/fped.2022.982179)
Supplement: Supplementary file 1 [file Table_1.DOCX]

**Search strategy**

### Database: Embase <to 2021 May 13>

#1: exp bacterial infection/

#2: (bacteria* or infect*).mp

#3: exp ertapenem/ or "etrapenem"/

#4: (ertapenem* or etrapenem* or invanoz or invanz or "l 749345" or "l-749345" or l749345 or "mk 0826" or "mk-0826" or "mk 826" or "mk-826" or mk0826 or mk826 or "zd 4433" or "zd-4433" or "zd4433").mp.

#5: (153773-82-1 or 153832-38-3 or 153832-46-3).rn

#6: (#3 or #4 or #5)

#7: exp pediatrics/

#8: (Infan* or newborn* or new-born* or perinat* or neonat* or baby or baby* or babies or toddler* or minor or minors* or boy or boys or boyfriend or boyhood or girl* or kid or kids or child* or children* or schoolchild* or school child* or adolescen* or juvenil* or youth* or teen* or under*age* or pubescen* or pediatric* or paediatric* or peadiatric* or school* or prematur* or preterm*).mp

#9: (#7 or #8)

#10: ((#6 and #9 and (#1 or #2))

### Database: PubMed <to 2021 May 14>

#1: (Bacterial Infections[MeSH Terms])

#2: bacteria* or infect*

#3: ertapenem* or etrapenem* or invanoz or invanz or "l 749345" or "l-749345" or l749345 or "mk 0826" or "mk-0826" or "mk 826" or "mk-826" or mk0826 or mk826 or "zd 4433" or "zd-4433" or "zd4433"

#4: 153773-82-1[Other Term] OR 153832-38-3[Other Term] OR 153832-46-3[Other Term]

#5: "ertapenem"[MeSH Terms] OR "ertapenem sodium"[MeSH Terms] OR etrapenem[MeSH Terms]

#6: (#3 OR #4 OR #5)

#7: "Pediatrics"[Mesh]

#8: (Infan* or newborn* or new-born* or perinat* or neonat* or baby or baby* or babies or toddler* or minor or minors* or boy or boys or boyfriend or boyhood or girl* or kid or kids or child* or children* or schoolchild* or school child* or adolescen* or juvenil* or youth* or teen* or under*age* or pubescen* or pediatric* or paediatric* or peadiatric* or school* or prematur* or preterm*)

#9: (#7 or #8)

#10: #6 and #9

### Database: Web of Science <to 2021 May 14>

#1: TS=(bacteria* OR infect*)

#2: WC=INFECTIOUS DISEASES

#3: ALL=(ertapenem* or etrapenem* or invanoz or invanz or "l 749345" or "l-749345" or l749345 or "mk 0826" or "mk-0826" or "mk 826" or "mk-826" or mk0826 or mk826 or "zd 4433" or "zd-4433" or "zd4433")

#4: ALL=("153773-82-1" OR "153832-38-3" OR "153832-46-3")

#5: #3 OR #4

#6: TS=(infan* or newborn* or new-born* or perinat* or neonat* or baby or baby* or babies or toddler* or minor or minors* or boy or boys or boyfriend or boyhood or girl* or kid or kids or child* or children* or schoolchild* or school child* or adolescen* or juvenil* or youth* or teen* or under*age* or pubescen* or pediatric* or paediatric* or peadiatric* or school* or prematur* or preterm*)

#7: WC=PEDIATRICS

#8: TS=HUMAN

#9: (#1 OR #2) AND #5 AND (#6 OR #7)

### Database: the Cochrane library <to 2021 May 14>

#1: MeSH descriptor: [Bacterial Infections] explode all trees

#2: bacteria* or infect*

#3: MeSH descriptor: [Ertapenem] explode all trees

#5: ("153773-82-1" or "153832-38-3" or "153832-46-3")

#6: MeSH descriptor: [Pediatrics] explode all trees

#7: (Infan* or newborn* or new-born* or perinat* or neonat* or baby or baby* or babies or toddler* or minor or minors* or boy or boys or boyfriend or boyhood or girl* or kid or kids or child* or children* or schoolchild* or school child* or adolescen* or juvenil* or youth* or teen* or under*age* or pubescen* or pediatric* or paediatric* or peadiatric* or school* or prematur* or preterm*)

#8: (#1 or #2)

#9: (#3 or #4 or #5)

#10: (#6 or #7)

#11: (#8 and #9 and #10)

### Database: CNKI (期刊、学位、会议，中英文扩展：否) <to 2021 May 14>

(SU%=厄他培南+厄他培南钠+怡万之 OR TKA=厄他培南+厄他培南钠+怡万之) AND (SU%=儿童+婴儿+幼儿+婴幼儿+少儿+小儿+学龄+学龄前+学生+小学生+早产儿+新生儿+儿科+低龄+适龄+患儿 OR TKA=儿童+婴儿+幼儿+婴幼儿+少儿+小儿+学龄+学龄前+学生+小学生+早产儿+新生儿+儿科+低龄+适龄+患儿)

### Database: 万方 (期刊、学位、会议) <to 2021 May 14>

主题:("厄他培南" OR "怡万之") and 主题:(儿童 OR 婴儿 OR 幼儿 OR 婴幼儿 OR 少儿 OR 小儿 OR 学龄 OR 学龄前 OR 学生 OR 小学生 OR 早产儿 OR 新生儿 OR 儿科 OR 低龄 OR 适龄 OR 患儿)

### Database: 维普 <to 2021 May 14>

(M=厄他培南+厄他培南钠+怡万之 OR R=厄他培南+厄他培南钠+怡万之) AND (M=儿童+婴儿+幼儿+婴幼儿+少儿+小儿+学龄+学龄前+学生+小学生+早产儿+新生儿+儿科+低龄+适龄+患儿 OR R=儿童+婴儿+幼儿+婴幼儿+少儿+小儿+学龄+学龄前+学生+小学生+早产儿+新生儿+儿科+低龄+适龄+患儿)

### Database: CBM <to 2021 May 14>

( "厄他培南"[常用字段:智能] OR "厄他培南钠"[常用字段:智能] OR "怡万之"[常用字段:智能]) AND ("儿童"[不加权:扩展] OR "婴儿"[不加权:扩展] OR "儿童"[常用字段:智能] OR "婴儿"[常用字段:智能] OR "幼儿"[常用字段:智能] OR "婴幼儿"[常用字段:智能] OR "少儿"[常用字段:智能] OR "小儿"[常用字段:智能] OR "学龄"[常用字段:智能] OR "学龄前"[常用字段:智能] OR "学生"[常用字段:智能] OR "小学生"[常用字段:智能] OR "早产儿"[常用字段:智能] OR "新生儿"[常用字段:智能] OR "儿科"[常用字段:智能] OR "低龄"[常用字段:智能] OR "适龄"[常用字段:智能] OR "患儿"[常用字段:智能])
