# Supplementary material for: Effectiveness of ertapenem for treatment of infections in children: An evidence mapping and meta-analysis
Source: Front Pediatr. 2022 Oct 12;10:982179. doi: 10.3389/fped.2022.982179 (PMC9620802; doi:10.3389/fped.2022.982179)
Supplement: Supplementary file 2 [file Table_2.DOCX]

**Appendix Table 1: Risk of bias assessment**

| Study ID | Overall | Randomization | Allocation concealment | Blinding of participants and personnel | Blinding of outcome assessor | Incomplete data | Selective reporting | Other bias |
| --- | --- | --- | --- | --- | --- | --- | --- | --- |
| Arguedas 2009 | High risk | Low risk | Moderate risk | Low risk | Moderate risk | Low risk | Moderate risk | High risk |
| Arnold 2018 | Moderate risk | Low risk | Low risk | Moderate risk | Moderate risk | Moderate risk | Moderate risk | Low risk |
| Dalgic 2014 | High risk | Low risk | Low risk | High risk | Moderate risk | Moderate risk | Moderate risk | Low risk |
| Jin 2013 | Moderate risk | Low risk | Moderate risk | Moderate risk | Moderate risk | Moderate risk | Moderate risk | Moderate risk |
| Pogorelic´ 2019 | Moderate risk | Low risk | Low risk | Moderate risk | Moderate risk | Moderate risk | Moderate risk | Low risk |
| Tang 2013 | Moderate risk | Low risk | Moderate risk | Moderate risk | Moderate risk | Moderate risk | Moderate risk | Low risk |
| Wirth 2018 | Moderate risk | Low risk | Moderate risk | Low risk | Moderate risk | Low risk | Moderate risk | Moderate risk |
| Yellin 2007 | Moderate risk | Low risk | Low risk | Low risk | Moderate risk | Low risk | Moderate risk | Moderate risk |
| Filip 2017 | 7 | * | * | * | - | ** | * | * |

Note: For Filip 2017 study, we evaluated the quality of studies using the Newcastle-Ottawa Quality Assessment Scale (NOS), an asterisk represented one point, total score was 7.
