# Supplementary material for: Effectiveness of ertapenem for treatment of infections in children: An evidence mapping and meta-analysis
Source: Front Pediatr. 2022 Oct 12;10:982179. doi: 10.3389/fped.2022.982179 (PMC9620802; doi:10.3389/fped.2022.982179)
Supplement: Supplementary file 3 [file Table_3.DOCX]

**Appendix Table 2: Summary of evidence type and regions**

|  | n of trials (%) | n of participants (%) |
| --- | --- | --- |
| **Study design** |  |  |
| RCT | 8 (53.3) | - |
| Observational comparative study | 1 (6.7) | - |
| Before-after study | 6 (40.0) | - |
| Total | 15 | - |
| **Region** |  |  |
| China | 3 (20.0) | 297 (11.8) |
| Croatia | 1 (6.7) | 80 (3.2) |
| German | 1 (6.7) | 451 (17.8) |
| Romania | 1 (6.7) | 96 (3.8) |
| Turkey | 3 (20.0) | 234 (9.3) |
| United States | 4 (26.7) | 854 (33.8) |
| United States, Spain, Mexico, Brazil | 2 (13.3) | 516 (20.4) |
| **Total** | 15 | 2528 |
