# Supplementary material for: Effectiveness of ertapenem for treatment of infections in children: An evidence mapping and meta-analysis
Source: Front Pediatr. 2022 Oct 12;10:982179. doi: 10.3389/fped.2022.982179 (PMC9620802; doi:10.3389/fped.2022.982179)
Supplement: Supplementary file 4 [file Table_4.DOCX]

**Appendix Table 3: Summary of findings for Ertapenem compared to Beta-lactam antibiotics for children infection**

| Outcomes | **Anticipated absolute effects (95% CI)** | | Relative effect (95% CI) | No. of participants (studies) | Certainty of the evidence (GRADE) |
| --- | --- | --- | --- | --- | --- |
|  | **Risk with Beta-lactam antibiotics** | **Risk with Ertapenem** |  |  |  |
| Treatment success rate | 847 per 1,000 | **915 per 1,000** (838 to 1,000) | **RR 1.08** (0.99 to 1.19) | 274 (3 RCTs) | ⨁⨁⨁◯ Moderate^a^ |
| Length of stay (days) | Mean length of stay was 7.55 | MD **0.44 higher** (0.5 lower to 1.38 higher) | - | 107 (1 RCT) | ⨁⨁◯◯ Low^b^ |
| Adverse event (any) | 146 per 1,000 | **149 per 1,000** (104 to 216) | **RR 1.02** (0.71 to 1.48) | 684 (4 RCTs) | ⨁⨁⨁◯ Moderate^a^ |
| Adverse events (diarrhea) | 89 per 1,000 | **55 per 1,000** (28 to 111) | **RR 0.62** (0.31 to 1.25) | 508 (2 RCTs) | ⨁⨁◯◯ Low^c^ |
| Adverse events (site pain) | 32 per 1,000 | **49 per 1,000** (5 to 457) | **RR 1.66** (0.59 to 4.68) | 508 (2 RCTs) | ⨁◯◯◯ Very low^c,d^ |

a. Downgraded by one level due to imprecision: wide confidence interval and low event rate.

b. Downgraded by two levels due to imprecision: wide confidence interval and very small sample size.

c. Downgraded by two levels due to imprecision: wide confidence interval and very low event rate.

d. Downgraded by one level due to unexplained inconsistency.
