# Supplementary figures and images for: Effectiveness of ertapenem for treatment of infections in children: An evidence mapping and meta-analysis
Source: Front Pediatr. 2022 Oct 12;10:982179. doi: 10.3389/fped.2022.982179 (PMC9620802; doi:10.3389/fped.2022.982179)

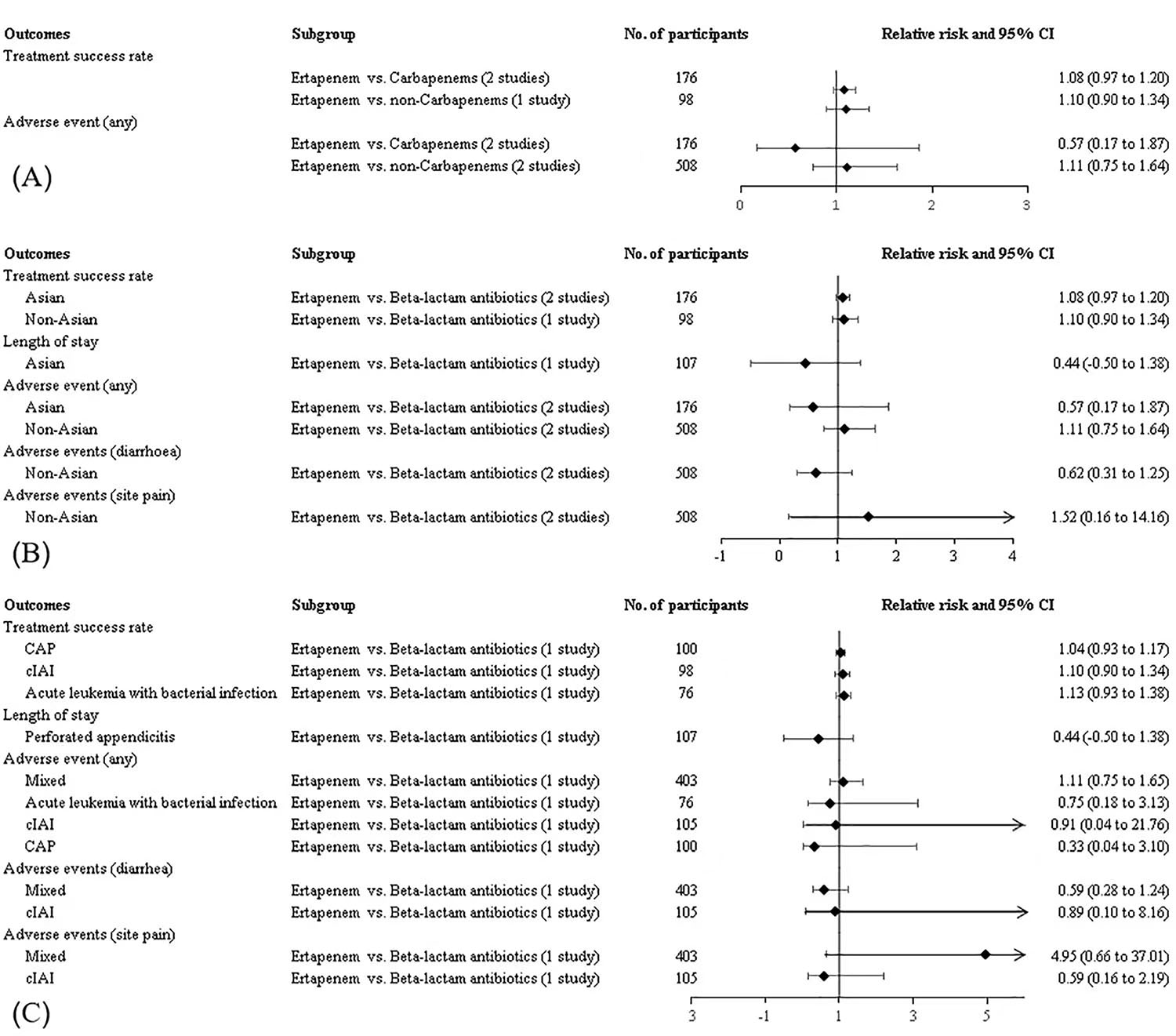

Supplement: Supplementary Appendix Figure 1 — Ertapenem vs. beta-lactam antibiotics (subgroup). (A) Ertapenem vs. carbapenems and ertapenem vs. non-carbapenems. (B) Asian vs. non-Asian patient population. (C) Different bacterial infection sites (cIAI and cUTI), post-infection cough, and CAP. [file Image_1.JPEG]
